# Pain management practice and associated factors among nurses working in Ethiopia: A systematic review and meta-analysis

**DOI:** 10.1371/journal.pone.0312499

**Published:** 2025-01-06

**Authors:** Melesse Abiye Munie, Amsalu Baylie Taye, Befkad Derese Tilahun, Addis Wondmagegn Alamaw, Gebremeskel Kibret Abebe, Migbaru Endawoke Tiruye, Biruk Beletew Abate

**Affiliations:** 1 Department of Nursing, College of Health Sciences, Woldia University, Woldia, Ethiopia; 2 Department of Emergency and Critical Care Nursing, School of Nursing, College of Medicine and Health Science, Woldia University, Woldia, Ethiopia; 3 Department of Nursing, College of Medicine and Health Science, Dilla University, Dilla, Ethiopia; 4 Department of Pediatrics Nursing, College of Health Sciences, Woldia University, Woldia, Ethiopia; Wollega University, ETHIOPIA

## Abstract

**Background:**

Pain management is a crucial component of patient care that promotes relaxation, lowers complications, improves quality of life, and shortens hospital stays. Several studies assessed the nurses’ pain management practices in Ethiopia. However, the findings of these studies are highly variable and inconsistent. Therefore, the study aims to determine the overall prevalence of pain management practice and associated factors among nurses working in Ethiopia.

**Method:**

The study included all observational quantitative research articles conducted among nurses in Ethiopia. We used Google Scholar, PubMed, Cochrane Library, and Scopus searching databases. The Newcastle—Ottawa Scale was used to check the study quality. Then I2 statistics and Cochran’s Q test were used to evaluate heterogeneity. The Funnel, Egger’s test, and non-parametric trim and fill effect tests were used to check publication bias by using a random effect model. Finally, we conducted subgroup analysis and sensitivity tests to evaluate statistical heterogeneity and the presence or absence of any influential study.

**Result:**

In the final analysis, we included eighteen studies, and 4,213 nurses participated. The overall pooled prevalence of nurses with good pain management practice was 43.79% (95% CI: 38.52, 49.06%). In-service training AOR; 95% CI 3.21 (1.87, 4.54), good knowledge AOR; 95% CI 2.44 (1.78, 3.09), positive attitudes AOR; 95% CI 2.84 (1.24, 4.44), and pain management guidelines in health facilities AOR; 95% CI 3.46 (1.48, 5.44) were the significant associated factors with pain management practice among nurses in Ethiopia.

**Conclusion:**

This study found that over half of Ethiopian nurses had poor pain management practices. Knowledge, attitude, training, and pain management guidelines in health facilities were significant factors. Therefore, health managers and stakeholders should prioritize pain management awareness, attitudes, availability of guidelines, and in-service training to improve patient care and outcomes.

## 1. Introduction

According to the International Association for the Study of Pain (IASP), ‘*pain is defined as an unpleasant sensory and emotional response to the potential and actual tissue damage* [1, 2]’. Pain is a major public health concern in both developed and developing countries. Globally, it has been estimated that one in five adults suffers from pain, and 1 in 10 adults is diagnosed with chronic pain each year. In Africa, the prevalence of pain is 87.5% in palliative care [3]. In Ethiopia, pain affects 73.1% of patients, and it is the leading anticipated problem in the postoperative period in Ethiopia [4].

Pain management is considered the fifth vital sign [5, 6] and constitutes an important area of health care and nursing care. It is necessary in terms of relaxing the individual, increasing the quality of life, reducing complications, and shortening the length of hospitalization [7]. Pain management is the alleviation of pain or a reduction in pain to a level of comfort that is acceptable to the client. It includes two basic types of nursing interventions: pharmacologic and non-pharmacologic [8, 9]. Pharmacological interventions consider drugs to treat and relieve pain. Non-pharmacological strategies such as physical therapy, occupational therapy, comfort therapy, psychosocial therapy, or counseling serve as an alternative treatment option in pain management [10, 11].

Untreated and undertreated pain significantly interferes with the patient’s physical, emotional, and spiritual well-being and also increases the incidence and severity of complications. Inadequately managed pain affects a patient’s quality of life, leading to a higher hospital readmission rate, more repeated outpatient visits, prolonged hospital stay, increased risk of nosocomial infection, impaired memory, drowsiness, tolerance, lack of concentration, and also increased stress and anxiety for the patient as well as his family. It also accounts for a high-cost problem in the healthcare economy [12, 13]. The financial expenditure related to the management of pain and opioid dependence is over 100 billion dollars per year [14].

Pain management is an important aspect of patient care that relaxes the individual, increases the quality of life, reduces complications, and shortens the length of hospitalization [7]. Although nurses play a key role in pain management, unfavorable attitudes and a lack of knowledge were reported as the major obstacles to implementing effective pain management practices among nurses [15] Globally, many studies have confirmed that a wide variety of factors affecting pain management, including insufficient education, a heavy workload, a lack of motivation, including salary, role confusion, and a lack of continuing training, contribute to inadequate pain management [16, 17].

Several studies conducted in Ethiopia assessed the nurses’ pain management practices. However, the findings of these studies are highly variable and inconsistent [18–22]. To our knowledge, until now there has been no systematic review and meta-analysis done to assess the nurses’ pain management practice and associated factors at a national level. Therefore, the purpose of this study is to determine the pooled prevalence of pain management practice and associated factors among Ethiopian nurses. The findings of this study will help policymakers, stakeholders, and other concerned bodies identify gaps and plan strategies to increase nurses’ pain management practice. Moreover, it will improve the quality of health care delivery by nurses by intervening in the gap accordingly.

## 2. Materials and methods

### 2.1 Study design and reporting

The study used asystematic review and meta-analysis to determine the pooled prevalence of nurses’ pain management practice and associated factors in Ethiopia. The study was conducted based on the Preferred Reporting Items for Systematic review and Meta-Analyses (PRISMA) guidelines recommendation [23] (S1 Table). The study had been registered on the International Prospective Register of Systematic Reviews (PROSPERO) protocol (CRD42024555769).

### 2.2 Inclusion and exclusion criteria

This study includes all original research articles conducted only in Ethiopia that fulfilled the inclusion criteria. We included the studies published only in English, used observational studies, had a quantitative research methodology, and were available in electronic resources from January 1, 2010, to December 30, 2023. In this study, articles that had no clearly stated outcome variable or no information on the practice outcome, studies conducted on health professionals other than nurses, low quality, and difficult to extract necessary information due to no full-text access were excluded.

### 2.3 Search strategy and source of information

The searching strategy was developed by using the adapted “PEOS” (population, exposure outcomes, study design, and setting) format for creating the MeSH terms to retrieve the potential studies in the database, as detailed below:

**Population**: Nurses
**Exposure**: pain management factors, associated factors, predictors, barriers, and determinants.
**Outcome**: Nurses’ pain management practice
**Study Design**: Observational Studies
**Setting (context)**: Ethiopia

To identify relevant primary studies, we developed the following review questions based on the above format:

“What is the national pooled prevalence of pain management practice among nurses in Ethiopia?”
“What factors affecting pain management practice among nurses in Ethiopia?”

Then, primary studies were searched using Google Scholar, PubMed/MEDLINE, Cochrane Library, and Scopus searching databases. The search was conducted from December 1 to December 30, 2023. The search was conducted in electronic databases using the following free-text terms: “pain management,” “practice,” “factors,” “associated factors,” “determinant factors,” “post-operative,” “critical care unit,” “admitted unit,” “pharmacological,” “non-pharmacological,” “nurses,” “hospital-based nurses,” and "Ethiopia." Items were searched using “AND” and “OR” Boolean operator strings. The published and unpublished articles from the Ethiopian university repository were also searched. After searching for accessible articles, all the retrieved articles were sorted, and the duplications were removed. The research encompassed studies on pain management practices and their significant factors among Ethiopian nurses.

### 2.4 Study selection

All retrieved articles were inserted into EndNote version 20, and duplicate files were eliminated from the analysis. Two investigators (MA and AB) independently screened articles based on the objective and form of the study. We reviewed the articles by title, abstract, and full text to identify potentially eligible studies according to the predetermined inclusion criteria. Then the screened articles were compiled together by the two reviewers.

### 2.5 Study quality assessment

To ensure the quality of each study we used a modified Newcastle—Ottawa Scale (NOS) by two independent reviewers [24]. The two reviewers (MA and AB) independently reviewed each of the articles that fulfilled the inclusion criteria and met the study’s objective. If any incongruities among the reviewers occurred, it was resolved by consulting other senior researchers (BB and BD). The assessment scale contains representativeness of the sample, sample size adequacy, the response rate or characteristics of responders and non-responders, measurement tools (ascertainment of exposure or risk factor), comparability of different outcome groups based on study design or analysis, controlled important and confounding factors, outcome assessment, and statistical tests. The assessment tool was measured out of 10 total scores. Studies with a score greater than five out of ten were considered to have a low risk of bias and included into the analysis. All included studies valued a score greater than 7 out of 10, indicating a low risk of bias (Table 1 and S2 Table).

**Table 1:** Descriptive summary of eighteen studies included in the meta-analysis of pain management practice and associated factors among nurses working in Ethiopia.

Author name	Year of publication.	Study design	Study area	Sample size	Sampling techniques	Good practice (%)	Working unit	Quality status
Wondimagegn ZG et al. [41]	2021	Cross-sectional	Addis Ababa	193	Non-random	56.5	ICU	Low risk
Tsegaye D. et al. [28]	2023	Cross-sectional	Amhara region	322	Random	48.1	All IPD and OPD	Low risk
Negewo AN et al. [32]	2020	Cross-sectional	Oromia region	187	Non-random	23.5	Post-operative	Low risk
Jalale Benti FL, and Hikma Shukre [29]	2021	Cross-sectional	Addis Ababa	169	Random	41	Post-operative	Low risk
Wari G. et al. [42]	2021	Cross-sectional	Addis Ababa	115	Random	32	ICU	Low risk
Jaleta D. et al. [46]	2020	Cross-sectional	Amhara region	405	Random	52.35	Post-operative	Low risk
Sharif Abdilahi A. [39]	2021	Cross-sectional	Somali region	110	Non-random	39.1	Post-operative	Low risk
Gimja Bitire W. [38]	2022	Cross-sectional	SNNP Region	276	Random	44.93	Post-operative	Low risk
Zeleke S. et al. [30]	2021	Cross-sectional	Amhara region	169	Non-random	26	All IPD and OPD	Low risk
Teshome ZB et al. [33]	2022	Cross-sectional	Oromia region	144	Random	44	Post-operative	Low risk
Kibret H. et al. [40]	2022	Cross-sectional	Harari and Dire Dawa	390	Random	54.1	All IPD and OPD	Low risk
Abdella Muhammed J. [34]	2021	Cross-sectional	Oromia region	418	Random	53.8	All IPD and OPD	Low risk
Mekonen MWM et al. [35]	2022	Cross-sectional	Oromia region	203	Random	33	ICU	Low risk
Feleke DG. et al. [31]	2024	Cross-sectional	Amhara region	283	Non-random	49.8	Post-operative	Low risk
Tadesse N. et al. [43]	2022	Cross-sectional	Addis Ababa	226	Non-random	38.1	Post-operative	Low risk
Wurjine TH, Nigussie BG. [36]	2018	Cross-sectional	Oromia region	144	Non-random	47.9	Post-operative	Low risk
Kassa RN, Kassa GM [44]	2014	Cross-sectional & qualitative	Addis Ababa	82	Non-random	34.1	All IPD and OPD	Low risk
Dechasa A. et al. [37]	2022	Cross-sectional	Oromia region	377	Random	66	Post-operative	Low risk

**Note**:—SNNPR: Southern Nations Nationalities and Peoples Region; IPD: inpatient department; OPD: outpatient department; ICU: intensive care unit; Low risk: a study scored > 50% in the NOS quality assessment scale.

### 2.6 Data extraction

The data extraction form was prepared on a Microsoft Excel spreadsheet. Two independent reviewers (MA and AB) extracted data from full-text articles. Then, we extracted data by using the data extraction form, which includes the first author name, publication year, setting (area), study design, sampling techniques, sample size, assessment tools, working unit, types of pain management (pharmacological, non-pharmacological, or both), study population, and number of cases. We used the prevalence for each first outcome and the adjusted odds ratio for each factor (second outcome) with a 95% confidence interval. Any discrepancies between the two data extractors were resolved by another author (BB and BD), which involved discussion and consensus (S3 and S4 Tables).

### 2.7 Outcome measurement

This study has two outcomes: the pooled prevalence of nurses’ pain management practices and significant associated factors. The level of nurses’ practice towards pain management was categorized as poor or good based on the practice assessment tool, which was measured using mean scores and different assessment scales. The factors affecting pain management practice were evaluated by their adjusted odds ratio and identified based on the statistical analysis results.

### 2.8 Statistical analysis

After the data extraction, we used the statistical software STATA version 17, and the meta-analysis was done. The prevalence estimates were conducted using the corresponding standard errors (SE), which were calculated using p = r/n and SE = √ p (1 − p)/n, respectively, where p is the proportion, r is the total number of nurses, and n is the sample size. The results of this meta-analysis were reported as the pooled prevalence of nurses’ pain management practices, accompanied by 95% confidence intervals (CIs). P-values less than 0.05 were considered indicative of statistical significance. We also identified the factors significantly associated with pain management practices among nurses by using STATA version 17. The random effect model was used in situations with substantial heterogeneity across the study [25]. The I2 index and Cochran’s Q test across the studies were used to evaluate heterogeneity. The I^2^ statistics estimate the observed differences between studies due to heterogeneity and ranged from 0 to 100%. The I^2^ value of 0% indicates the absence of heterogeneity, whereas a value of 100% indicates significant heterogeneity. I^2^ statistics below 25% indicated low heterogeneity, between 25% and 50% moderate heterogeneity, and over 75% high heterogeneity [26]. Because the test statistic indicated significant heterogeneity among studies (I^2^ > 75% and p < 0.05), a random effect model was used to evaluate the level of nurses’ pain management practice with a 95% CI. To decrease the random variations between the primary studies, a subgroup analysis was conducted to determine the practice of nurses’ pain management across the different hospitals in the country. To check publication bias funnel plots for asymmetry, Egger’s test and non-parametric trim and fill effect tests were used. Although both the funnel plot and Egger’s test were employed to detect publication bias, Egger’s test is more effective than the funnel plot. The funnel plot shows the subjective view of asymmetry, whereas Egger’s test displays the actual values of effect sizes and their precision [27]. A sensitivity analysis was conducted to assess the robustness of the synthesized results. To handle missing data we used the PRISMA checklist that contained key items including the description of the included primary studies, the risk of bias assessment, and how missing was data handled. A sensitivity analysis was also conducted to assess how missing data from the study might influence the results and to ensure the robustness of our findings.

### 2.9 Ethical approval and consent from participants

This is not applicable because no primary data was collected from the participants.

## 3. Results

### 3.1 Literature search

**Fig 1:**
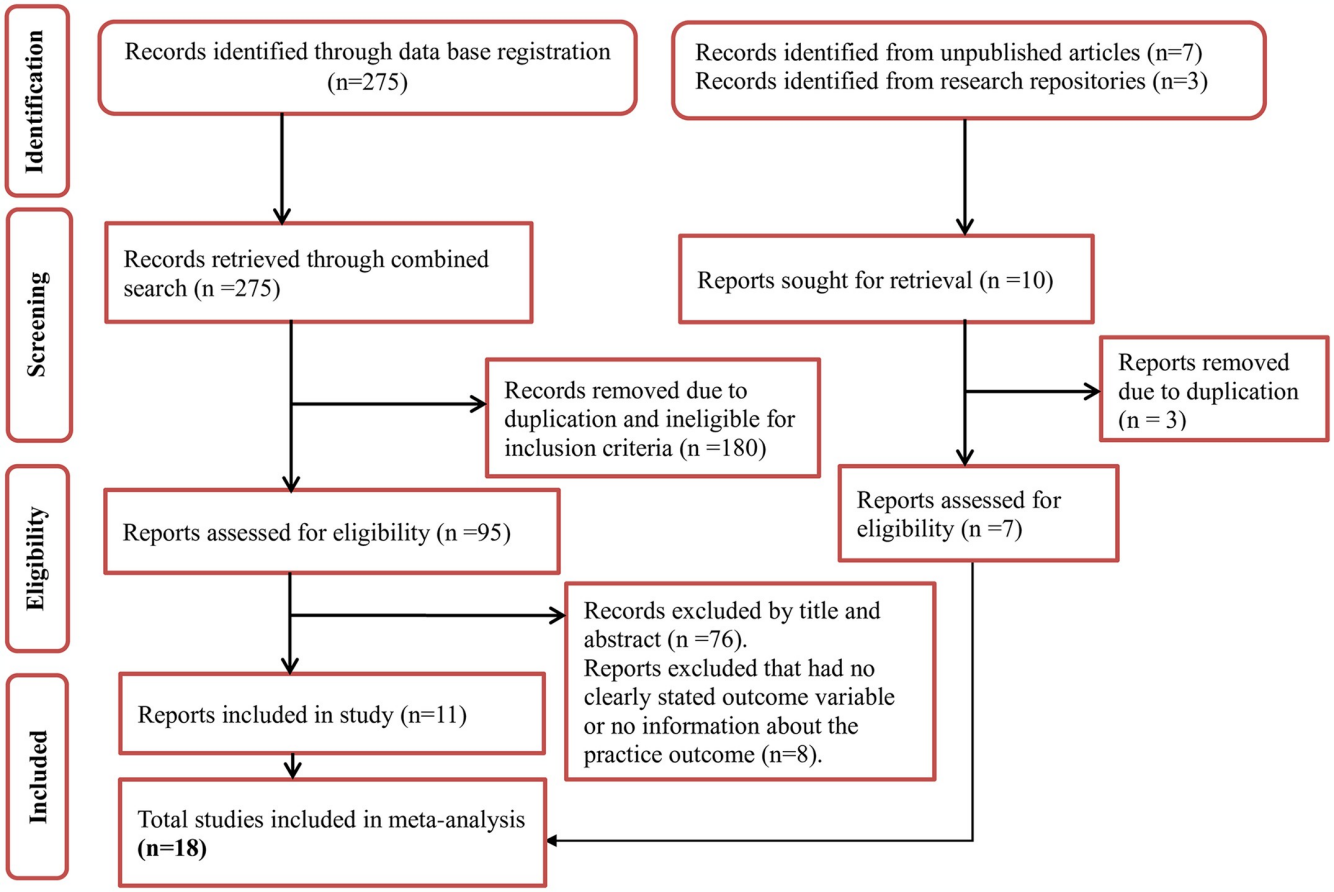
Flow chart of selection for systematic review and meta-analysis on the pain management practice and associated factors among nurses working in Ethiopia.

In the initial search, 285 published and unpublished records were retrieved, of which 180 published and 3 unpublished articles were omitted from the study due to duplicates and ineligible inclusion criteria. After carefully examining the abstracts and titles of the remaining 102 records, 84 of the reports from the published articles were further excluded. The final analysis contained 18 studies (Fig 1).

### 3.2 Study characteristics

All articles included in this systematic review and meta-analysis utilized a cross-sectional study design to assess nurses’ pain management practices. Overall information regarding the prevalence of pain management practice was obtained from four regions, including the Amhara region [28–31], Oromia region [32–37], Southern Nation, Nationalities, and Peoples (SNNP) region [38], Somalia region [39], and two self-administrative cities, including Dire Dawa [40] and Addis Ababa [41–45]. The sample size for this study ranged from 82 [44] to 422 [34] (Table 1).

### 3.3 Publication bias

**Fig 2:**
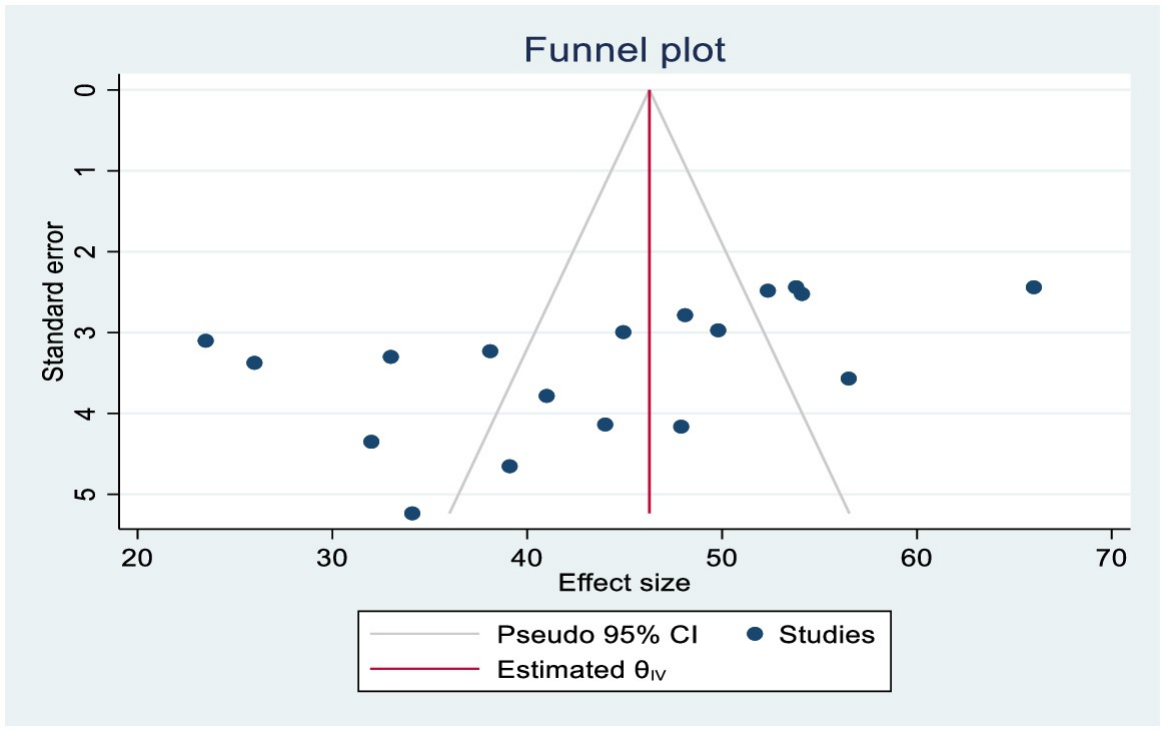
Funnel plot with 95% confidence interval of the pooled prevalence of nurses’ practice about pain management and associated factors in Ethiopia.

**Fig 3:**
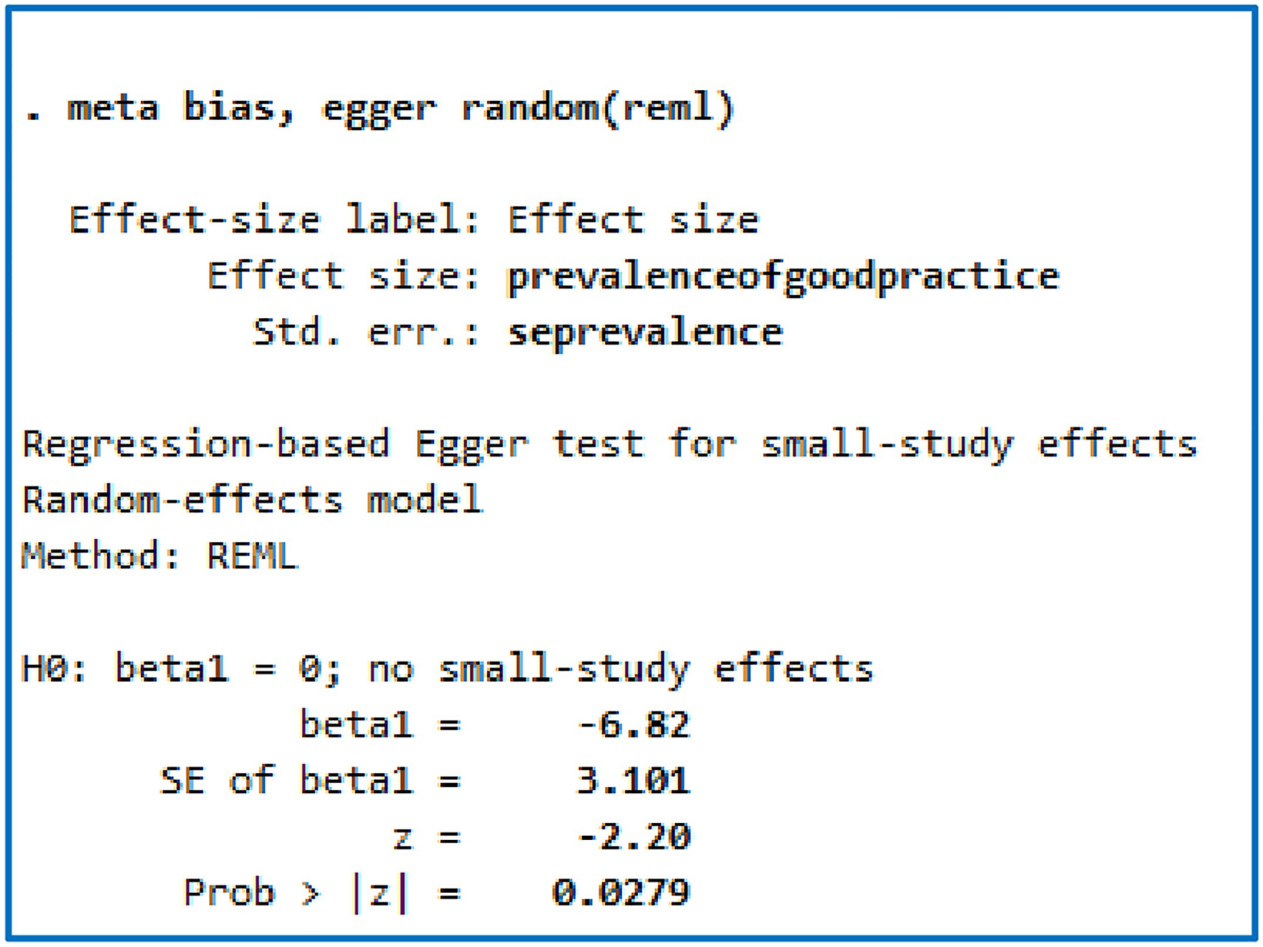
This figure shows the result of Egger test statistically significant using the random-effect analysis.

**Fig 4:**
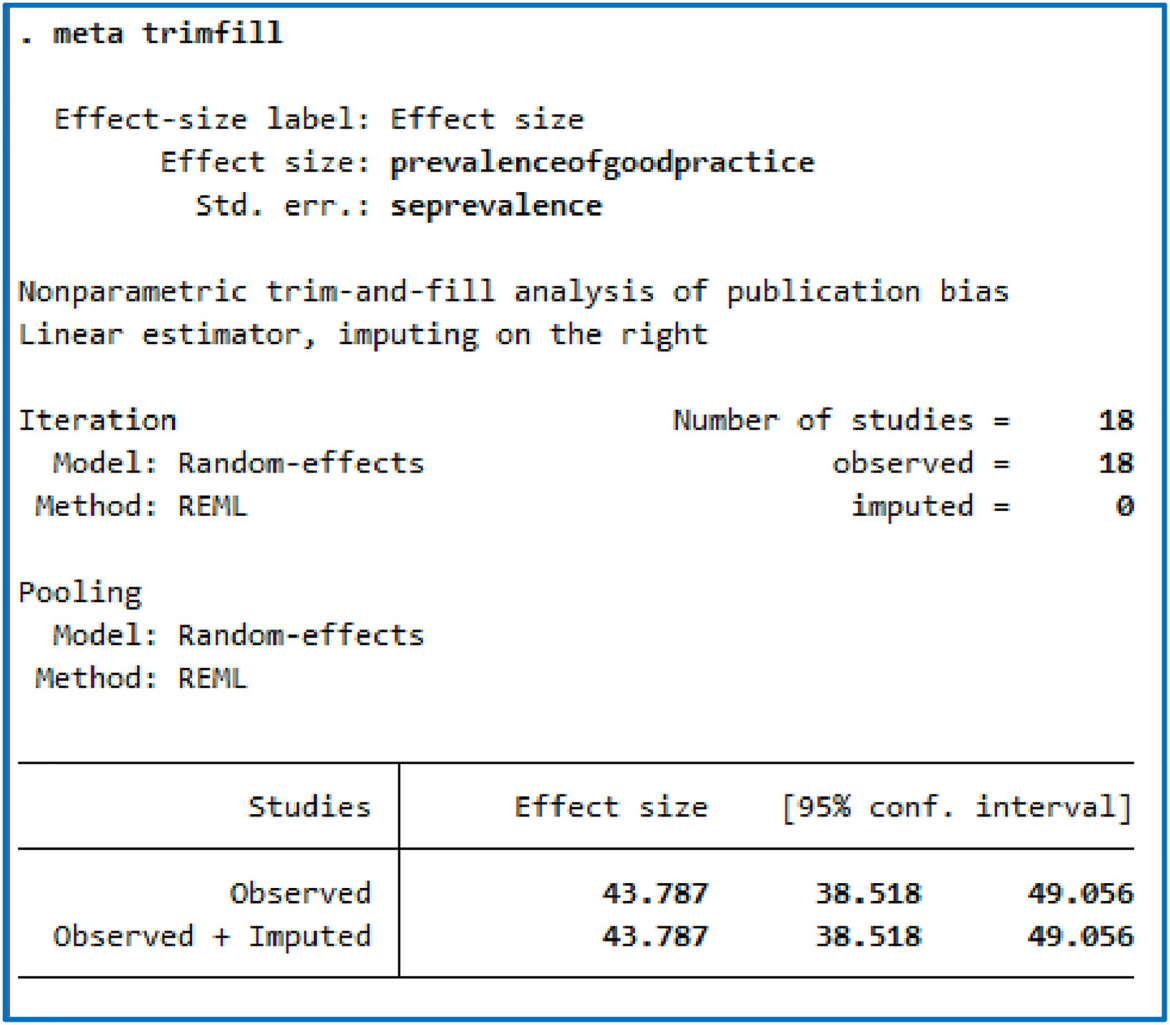
This figure shows the result of non-parametric trim and fills analysis of the pooled prevalence of nurses’ practice of pain management and associated factors in Ethiopia using the random-effect analysis.

In this meta-analysis, funnel plots and Egger’s test were conducted to check the presence of publication bias. The funnel plot revealed an asymmetrical distribution of studies (Fig 2). The result of the Egger test was also statistically significant, suggesting publication bias (p = 0.0279) (Fig 3). Therefore, we conducted a non-parametric trim and fill analysis to investigate potentially missing studies due to publication bias in the funnel plot and adjust the overall effect estimate. The analysis revealed no imputed missing studies, and the pooled prevalence of pain management practice was 43.787 (95% CI: 38.518, 49.056) (Fig 4).

### 3.4 Pooled prevalence of nurses’ pain management practice

**Fig 5:**
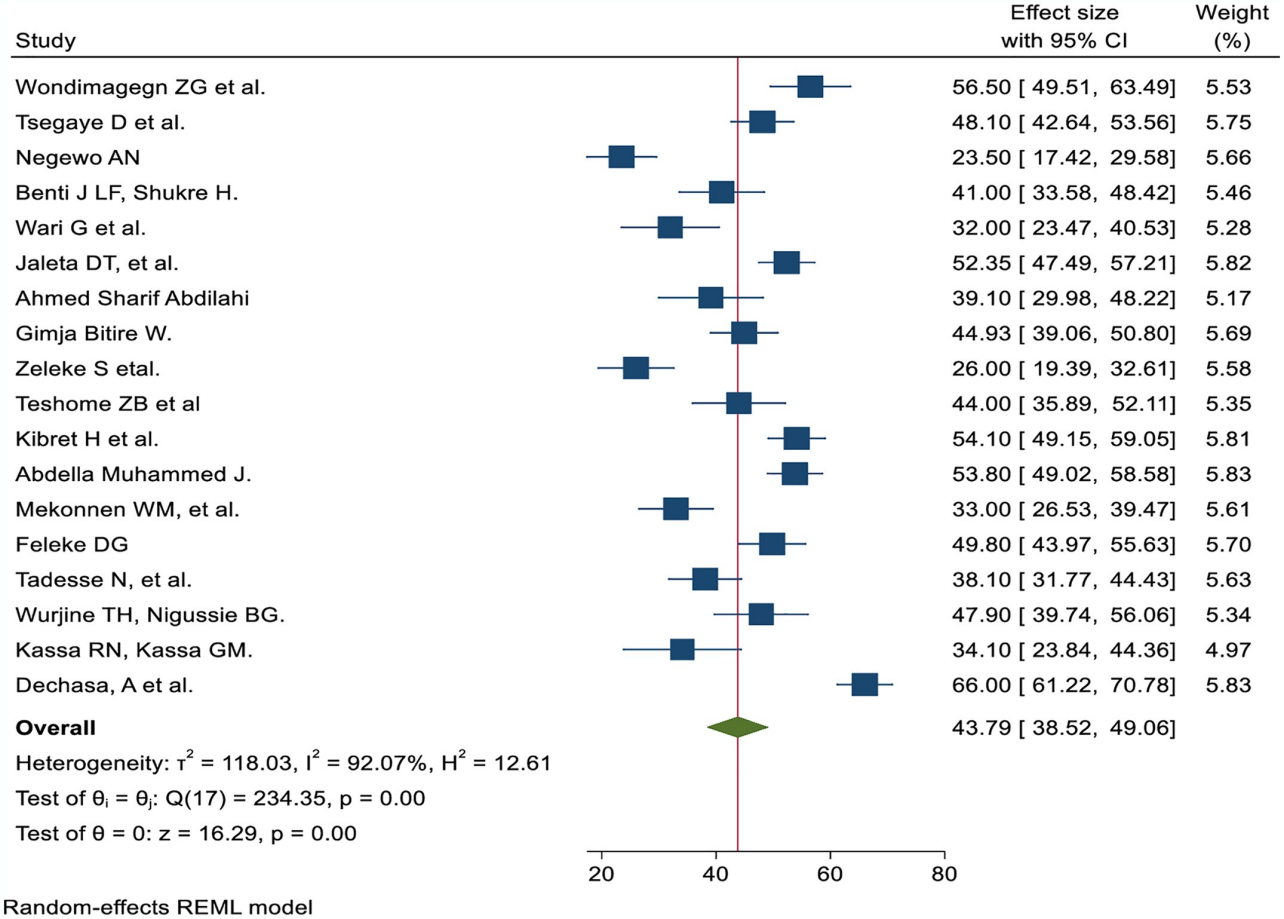
The pooled prevalence of good practice of pain management among nurse works in Ethiopia by using the random effect model.

In this study, 18 studies were used, and 4,213 nurses participated. The overall prevalence of good pain management practice among nurses was 43.79% (95% CI; 38.52, 49.06%) using random effect models (I^2^ = 92.07%, p = 0.000) (Fig 5).

### 3.5 Subgroup analysis

Due to heterogeneity, we performed a subgroup analysis based on the study area, year of publication, sampling techniques, sample size, working units, and types of management (pharmacological, non-pharmacological, and both). Based on the study area, the prevalence was higher in Dire Dawa and Harari (54.10% (95% CI: 49.15, 59.05, I^2^ = 0.00, p = 0.00) than in the Somali region (39.10% (95% CI: 29.98, 48.22, I^2^ = 0.000, p = 0.00) (S1 Fig). Based on the year of publication, the study done in the year greater than 2020 showed a higher prevalence of good pain management practice (44.07 (95% CI: 38.30, 49.83, I^2^ = 92.07%, p = 0.00) than in the year less than 2019 (41.37 (95% CI: 27.86, 54.87, I^2^ = 76.51%, p = 0.00) (S2 Fig). Based on sample size, there was a higher prevalence of good pain management practice in the studies’ sample size above 300 (54.12 (95% CI: 49.03, 59.21, I^2^ = 83.37%, p = 0.00) than the sample size below 300 (38.31 (95% CI: 32.88, 43.74, I2 = 85.04%, p = 0.00) (S3 Fig). Based on the sampling techniques, there was a higher prevalence of good pain management practice in the study done by random sampling techniques (47.25 (95% CI: 40.86, 53.63, I2 = 92.07%, p = 0.00) than non-random sampling techniques (39.38 (95% CI: 31.20, 47.55, I2 = 90.47%, p = 0.00) (S4 Fig). According to the study working unit, nurses working in the post-operative unit have a higher prevalence of good pain management practice (44.81 (95% CI: 37.80, 51.82, I^2^ = 91.83%, p = 0.00) than in the ICU 40.56 (95% CI: 24.83, 56.30, I^2^ = 92.87%, p = 0.00) (S5 Fig). Based on the type of management, nurses who practiced non-pharmacological management of pain had a higher prevalence of good practice (46.50 (95% CI: 36.44, 56.56, I2 = 94.13 9%, p = 000) than pharmacological pain management practice (39.95 (95% CI: 7.61, 72.29, I^2^ = 97.95%, p = 0.00) (S6 Fig).

### 3.6 Sensitivity analysis

Sensitivity analysis was performed to examine the effects of different outlying studies or any influential study on the pooled prevalence estimate of nurses’ pain management practice. The sensitivity analysis using a random effect showed that no study had a significant influence on the results, as all point estimates fell within the 95% confidence interval (Table 2).

**Table 2:** The result of the sensitivity analysis conducted on the prevalence of nurses’ practices about pain management and associated factors in Ethiopia.

Author name	Year of publication.	Point estimated	95%Confidence Interval
Wondimagegn ZG et al.	2021	43.02	37.32–48.72
Tsegaye D et al.	2023	43.49	37.60–49.38
Negewo AN et al.	2020	45.04	39.94–50.14
Jalale Benti FL, & Hikma Shukre	2021	43.92	38.16–49.69
Wari G et al.	2021	44.43	38.81–50.05
Jaleta D, et al.	2020	43.23	37.36–49.10
Sharif Abdilahi A.	2021	44.02	38.31–49.73
Gimja Bitire W.	2022	43.69	37.82–49.55
Zeleke S, et al.	2021	44.45	39.51–50.18
Teshome ZB et al.	2022	43.75	37.98–49.51
Kibret H et al.	2022	43.13	37.32–48.93
Abdella Muhammed J.	2021	43.14	37.32–48.96
Mekonen MWM et al.	2022	44.41	38.79–50.03
Feleke DG et al.	2024	43.39	37.54–49.24
Tadesse N et al.	2022	44.10	38.35–49.85
Wurjine TH, Nigussie BG.	2018	43.53	37.78–49.29
Kassa RN, Kassa GM.	2014	44.28	38.62–49.94
Dechasa A et al.	2022	42.44	37.47–47.41
**Combined**		**43.79**	**38.52–49.06**

### 3.7 Factors affecting nurses’ pain management practice in Ethiopia

**Fig 6:**
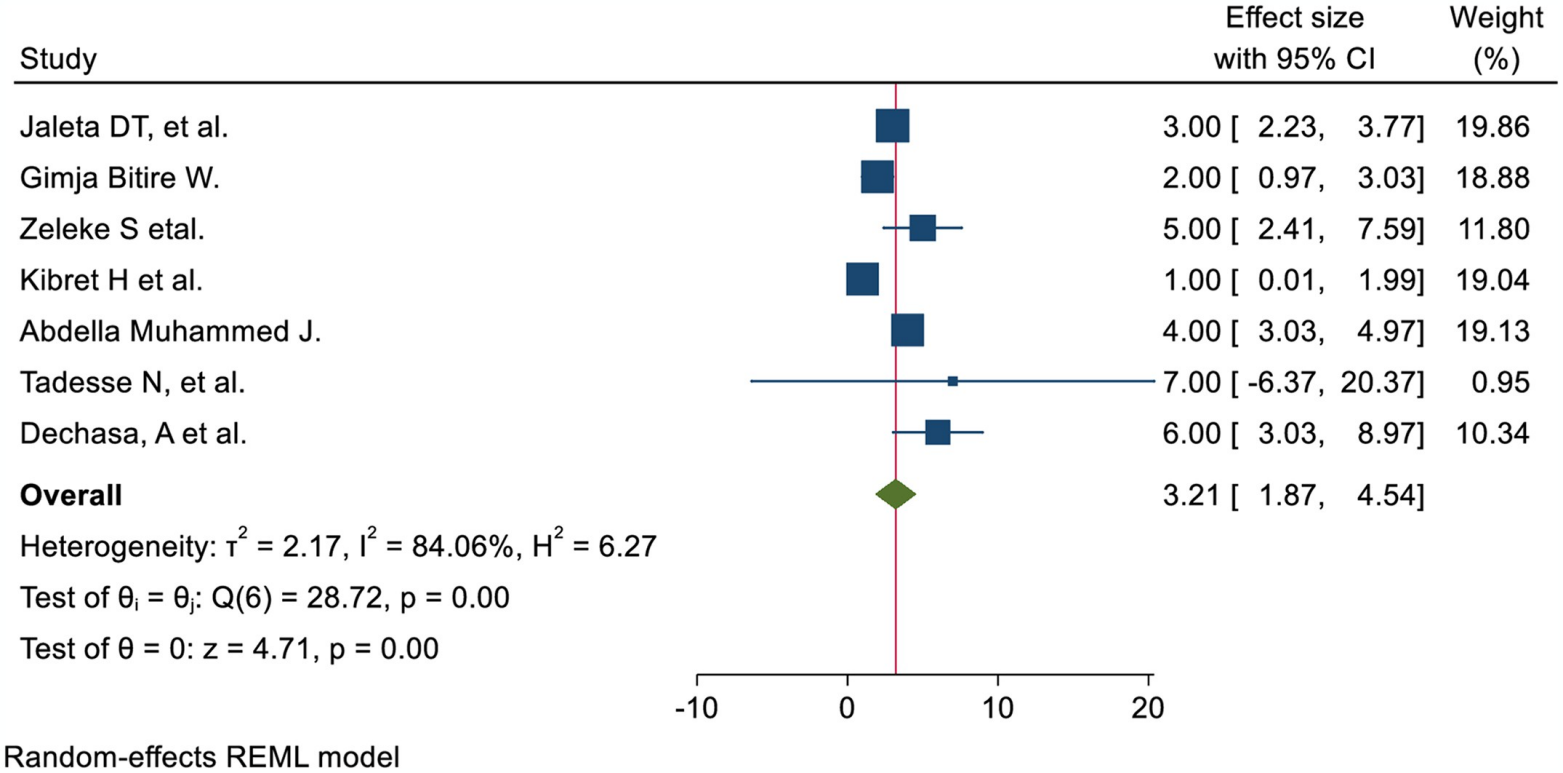
Forest plot shows the association between in-service training and pain management practice among nurses in Ethiopia.

**Fig 7:**
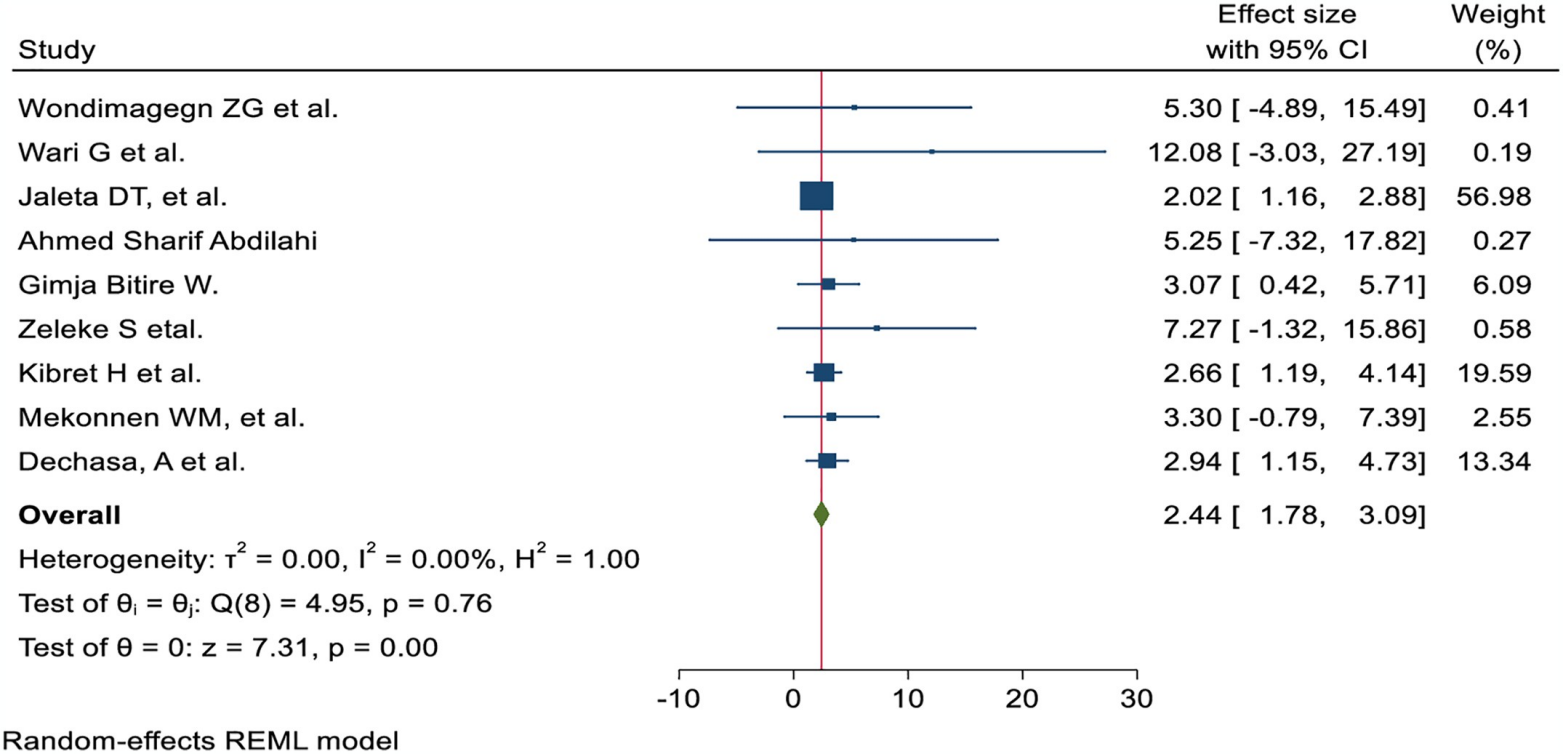
Forest plot shows the association between good knowledge and pain management practice among nurses in Ethiopia.

**Fig 8:**
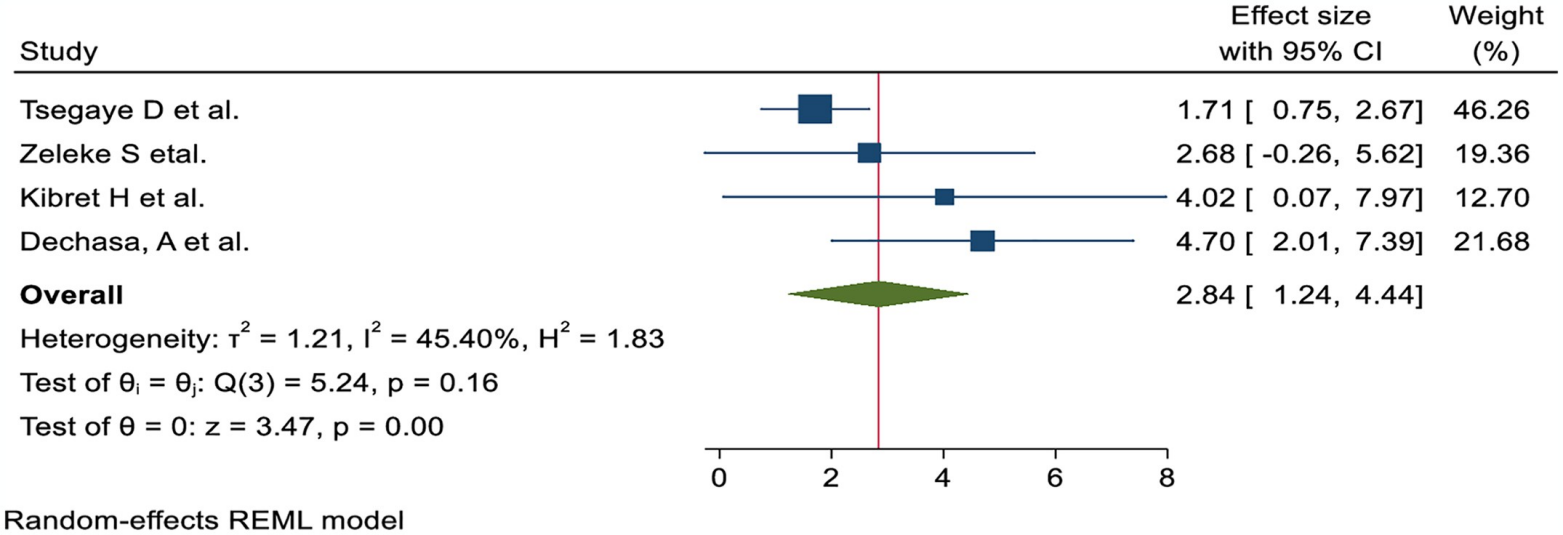
Forest plot shows the association between positive attitudes and pain management practice among nurses in Ethiopia.

**Fig 9:**
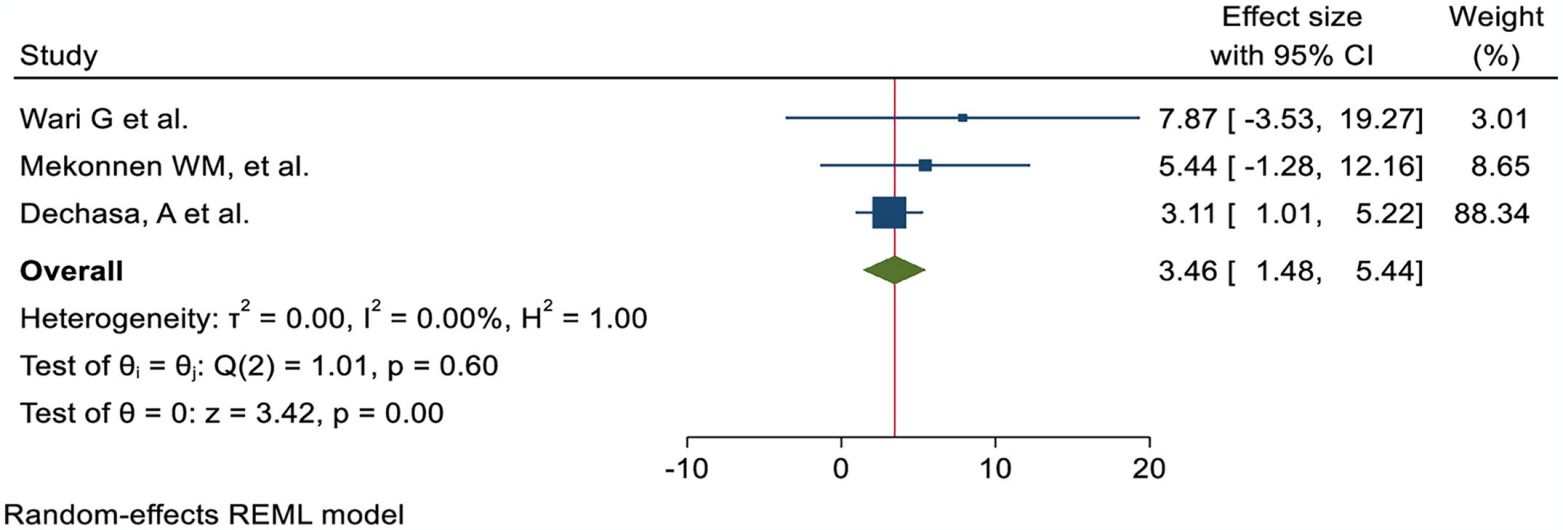
Forest plot shows the association between pain management guideline in health facility and pain management practice among nurses in Ethiopia.

The nurses’ pain management practice was 3.21 times more likely to have good practice of pain management among nurses trained in pain management than those who were not AOR; 95% CI 3.21 (1.87, 4.54) (Fig 6). Nurses with high knowledge of pain management were 2.44 times more likely to demonstrate it than their counterparts (AOR; 95% CI 2.44 (1.78, 3.09)) (Fig 7). The odds of nurses practicing pain management were 2.84 times higher among those who had favorable attitudes toward pain management than their counterparts (AOR; 95% CI 2.84 (1.24, 4.44) (Fig 8). Nurses who had access to pain management guidelines at their healthcare facilities were 3.46 times more likely to demonstrate good pain management practice than those who did not have such guidelines available (AOR; 95% CI 3.46 (1.48, 5.44) (Fig 9).

## 4. Discussion

The main objective of this systematic review and meta-analysis study was to estimate the overall pain management practice and its significant associated factors among nurses working in Ethiopia. The purposes of this study are to give insight into the current ways of managing pain and inform future practice to promote patient well-being and healing. The meta-analysis found that 43.79% of Ethiopian nurses practiced good pain management (95% CI: 38.12, 49.06%). This finding was lower than the research conducted in Iran [47] (55%), Turkey [48] (62.4%), Norway [49] (59%), Nigeria [50] (70.8%), Rwanda [51] (88%), and Jordan [52] (69%). The discrepancy might be due to the variety in sample size, system-related barriers like patient-to-nurse ratio, the study setting, methods, level of education, education about pain management, training on pain management, and attitudes toward pain management. The finding was higher than the studies conducted in Zagazig University Hospitals [53] (32.7%) and Nyanza and Western Kenya nurses practiced pain management in neonates through the use of non-pharmacologic (34.8%) and pharmacologic (28.1%) strategies [54]. The discrepancy might be related to the working unit, types of pain management, sample size, methods, socioeconomic status, and setting.

Subgroup analysis was conducted using the study area, year of publication, sample size, sampling techniques, working unit, and type of pain management. Based on the study area, the result found that pain management practices were more prevalent in Dire Dawa and Harari than in Somalia. This might be due to a few studies being conducted in this region than in others. According to the year the studies were published, the studies conducted after 2020 had a higher prevalence of good pain management practices than studies conducted before 2019. This variation might be attributed to nurses becoming more aware, trained, and having more positive attitudes towards pain management. The studies with a sample size of 300 or greater had a higher prevalence of good pain management practices than studies with a sample size of less than 300. This could be because the smaller sample size of the studies may have had lower reliability and generalizability. Based on the sampling techniques used, studies that employed random sampling had a higher prevalence of good pain management practices than studies that used non-random sampling methods. This could be because random sampling can reduce bias and increase representativeness for the general population. The study found that nurses who worked in the post-operative unit demonstrated a higher prevalence of effective pain management practices than in the intensive care unit (ICU). This discrepancy might be due to several reasons, such as the nurses’ workload, lack of knowledge and attitudes toward the assessment and management of pain, lack of communication between nurses and patients, inadequate training given for the management of critically ill patients, and lack of awareness on the effect of pain on patient outcome. Based on the type of pain management practice, nurses who perform non-pharmacological pain management had a higher prevalence of good practice than pharmacological pain management. The possible explanation could be that the nurses preferred non-pharmacological pain management because it offers a safer, more holistic, and patient-centered approach compared to relying solely on medications.

Those nurses who received pain management training were 3.21 times more likely to have effective pain management practices than those who did not AOR; 95% CI 3.21 (1.87, 4.54). This finding is strongly supported by the studies conducted at Jordan University [55] and Saudi Arabia [56]. The possible explanation might be that in-service training on pain management increasing nurses’ knowledge, comprehension of patients’ suffering, competency, and professional skills, leading to an improvement in the standard of pain management practices. Nurses with adequate understanding of pain management practice were 2.44 times more likely to practice effectively than those with insufficient knowledge AOR; 95% CI 2.44 (1.78, 3.09). This finding is corroborated by the studies conducted in Egypt [57], Taiwan [58], Canada [59], and Jordan University [55]. A possible explanation could be that the nurses’ level of understanding increases as their level of practice improves. Therefore, efficient knowledge is required to enhance the role of nurses in pain management practices, including patient advocacy, pain assessment, management of pain, and evaluation of the outcome. Those nurses who had favorable attitudes were 2.84 times more likely to have effective pain management practice than those who had no AOR; 95% CI 2.84 (1.24, 4.44). The findings of this study were supported by the studies conducted in Canada [59] and Jordan University [55]. A possible explanation could be that positive perceptions of pain management among nurses may have contributed to effective practice. Those nurses who followed a pain management guideline in a health facility were 3.46 times more likely to conduct good pain management practices than their counterparts AOR; 95%CI 3.46 (1.48, 5.44). The finding was in line with the results from the studies carried out in Nigeria [50] and Poland [60]. A possible justification could be that the availability of pain management guidelines may improve nurses’ knowledge and attitudes to enhance effective pain management practice.

Even though the study was not done without limitation, it has strengths that covered a broad range of articles, making the findings more comprehensive and accurate. Additionally, the researchers conducted subgroup and sensitivity analyses to address heterogeneity and ensure the robustness of the influential studies included. However, the study had some limitations. It only included cross-sectional studies, which may be limited in establishing causal relationships. The study also relied heavily on self-reported data from respondents, which could be subject to recall bias and may not accurately reflect the actual practices of the participants. Furthermore, the study used different measurement methods for the outcome variables, such as using the mean or different scales, which could make it challenging to draw definitive conclusions. Besides this, only published studies in the English language were included which might have bias and some of the studies did not report an appropriate randomization process.

## 5. Conclusions and recommendations

The study found that more than half of Ethiopian nurses had poor pain management practices. Knowledge, attitudes, in-service training, and pain management guidelines were the significant factors affecting pain management practice. Therefore, to enhance the quality of patient care and outcomes, health managers and stakeholders should prioritize initiatives that increase awareness, foster positive attitudes, develop comprehensive pain management guidelines, and provide targeted training for nurses in this critical area.

### 5.1 Implication of the study for policymakers

We urge policymakers to adopt pain as a fifth vital sign and integrate it into the healthcare system. It is essential to incorporate pain management education into nursing curricula, offers substantial training to improve nurses’ knowledge and attitudes toward pain management, and establish standardized pain management guidelines. Addressing these issues is crucial for advancing healthcare quality in Ethiopia, and we hope our findings will inform future policies and practices in pain management.

## Supporting information

S1 Table This table shows the PRISMA 2020 checklist followed for this systematic review and meta-analysis. (DOCX)

S2 Table This table shows the quality assessment of cross-sectional studies using the modified Newcastle—Ottawa Scale (NOS). (DOCX)

S3 Table This table shows the data extraction format for included studies developed by two independent reviewers in consciences. (XLSX)

S4 Table This table shows the data extraction format for all studies identified in the literature search, including those that were excluded from the analyses. (XLSX)

S1 Fig This is the subgroup analysis based on the study area of the pooled prevalence of nurses’ pain management practice in Ethiopia. (DOCX)

S2 Fig This is the subgroup analysis based on the year of publication for the pooled prevalence of nurses’ pain management practice and associated factors in Ethiopia. (DOCX)

S3 Fig This is the subgroup analysis based on the sample size for the pooled prevalence of nurses’ pain management practice in Ethiopia. (DOCX)

S4 Fig This is the subgroup analysis based on the sampling techniques for the pooled prevalence of nurses’ pain. (DOCX)

S5 Fig This is the subgroup analysis based on the working unit for the pooled prevalence of nurses’ pain management practice in Ethiopia. (DOCX)

S6 Fig This is the subgroup analysis based on the type of pain management for the pooled prevalence of nurses’ pain management practice in Ethiopia. (DOCX)

## Abbreviations

AOR: Adjusted Odds Ratio
CI: Confidence Interval
ICU: Intensive Care Unit
IPD: Inpatient Department
OPD: Outpatient Department
PRISMA: Preferred Reporting Items for Systematic Review and Meta-analysis
