# Supplementary material for: Pain management practice and associated factors among nurses working in Ethiopia: A systematic review and meta-analysis
Source: PLoS One. 2025 Jan 6;20(1):e0312499. doi: 10.1371/journal.pone.0312499 (PMC11703039; doi:10.1371/journal.pone.0312499)
Supplement: S2 Table — (DOCX) [file pone.0312499.s002.docx]

Supplementary Table 2: Methodological quality assessment of cross-sectional studies using modified Newcastle - Ottawa Scale (NOS)

| **First author, publication year** | Criteria | | | | | | | |  |  |
| --- | --- | --- | --- | --- | --- | --- | --- | --- | --- | --- |
|  | **Selection** | | | | **Comparability** | | **Outcome** | |  |  |
|  | Representativeness of the sample | Sample size | Non –respondents | Ascertainment of exposure/risk factor | Study controls most important factor | Study controls any additional factor | Assessment of the outcome | Statistical test | Total score  (10) | Overall quality |
| Wondimagegn ZG et al. 2021 | **B*** | **A*** | **A*** | **B*** | **A*** | **B*** | C* | **A*** | **8** | Low risk |
| Tsegaye D et al. 2023 | **A*** | **A*** | **A*** | **B*** | **A*** | **B*** | B** | **A*** | **9** | Low risk |
| Negewo AN, 2020 | **B*** | **A*** | **A*** | **A**** | **A*** |  | C* | **B** | **7** | Low risk |
| Benti J LF, Shukre H. 2021 | **A*** | **A*** | **C** | **B*** | **A*** | **B*** | A** | **A*** | **8** | Low risk |
| Wari G et al. 2021 | **A*** | **A*** | **A*** | **A**** | **A*** | **B*** | C* | **A*** | **9** | Low risk |
| Jaleta DT, et al. 2020 | **A*** | **A*** | **A*** | **B*** | **A*** | **B*** | B** | **A*** | **9** | Low risk |
| Ahmed Sharif Abdilahi, 2021 | A* | **A*** | **A*** | **B*** | **A*** | **B*** | A** | **A*** | **9** | Low risk |
| Gimja Bitire W. 2022 | A* | **A*** | **A*** | **B*** | **A*** | **B*** | A** | **A*** | **9** | Low risk |
| Zeleke S etal. 2021 | A* | A* | A* | B* | A* | B* | B** | A* | 9 | Low risk |
| Teshome ZB et al. 2022 | A* | A* | A* | B* | A* |  | C* | A* | 7 | Low risk |
| Kibret H et al. 2020 | A* | A* | A* | B* | A* | B* | A** | A* | 9 | Low risk |
| Abdella Muhammed J. 2021 | A* | A* | A* | B* | A* | B* | A** | A* | 9 | Low risk |
| Mekonnen WM, et al. 2022 | A* | A* | A* | B* | A* | B* | C* | A* | 8 | Low risk |
| Feleke DG, 2024 | B* | A* | C | B* | A* |  | A** | A* | 7 | Low risk |
| Tadesse N, et al. 2022 | B* | A* | A* | A** | A* | B* | A** | A* | 10 | Low risk |
| Wurjine TH, Nigussie BG. 2018 | B* | A* | A* | B* | A* |  | A** |  | 7 | Low risk |
| Kassa RN, Kassa GM. 2014 | C | A* | A* | A** | A* | B* | A** | A* | 9 | Low risk |
| Dechasa, A et al. 2022 | A* | A* | A* | B* | A* | B* | A** | A* | 9 | Low risk |

*Note: from each item account point. (Accept the study if total score ≥5)*

NEWCASTLE - OTTAWA QUALITY ASSESSMENT SCALE (adapted for cross sectional studies)

Selection: (Maximum 5 stars)

1) Representativeness of the sample:

a) Truly representative of the average in the target population. * (all subjects or random sampling)

b) Somewhat representative of the average in the target population. * (nonrandom sampling)

c) Selected group of users.

d) No description of the sampling strategy.

2) Sample size:

a) Justified and satisfactory. *

b) Not justified.

3) Non-respondents:

a) Comparability between respondents and non-respondents characteristics is established, and the response rate is satisfactory. *

b) The response rate is unsatisfactory, or the comparability between respondents and non-respondents is unsatisfactory.

c) No description of the response rate or the characteristics of the responders and the non-responders.

4) Ascertainment of the exposure (risk factor):

a) Validated measurement tool. **

b) Non-validated measurement tool, but the tool is available or described.*

c) No description of the measurement tool.

**Comparability: (Maximum 2 stars)**

1) The subjects in different outcome groups are comparable, based on the study design or analysis. Confounding factors are controlled.

a) The study controls for the most important factor (select one). *

b) The study control for any additional factor. *

**Outcome: (Maximum 3 stars)**

1) Assessment of the outcome:

a) Independent blind assessment. **

b) Record linkage. **

c) Self report. *

d) No description.

2) Statistical test:

a) The statistical test used to analyze the data is clearly described and appropriate, and the measurement of the association is presented, including confidence intervals and the probability level (p value). *

b) The statistical test is not appropriate, not described or incomplete.

This scale has been adapted from the Newcastle-Ottawa Quality Assessment Scale for non-randomized control studies to perform a quality assessment of cross-sectional studies for the systematic review.
