# Supplementary material for: Pain management practice and associated factors among nurses working in Ethiopia: A systematic review and meta-analysis
Source: PLoS One. 2025 Jan 6;20(1):e0312499. doi: 10.1371/journal.pone.0312499 (PMC11703039; doi:10.1371/journal.pone.0312499)
Supplement: S2 Fig — (DOCX) [file pone.0312499.s006.docx]

S1 Figure 2: this is the subgroup analysis based on year of publication for the pooled prevalence of nurses’ pain management practice and associated factors in Ethiopia.
